# Principles and Strategies of Interviewing People with Advanced Dementia

**DOI:** 10.1177/14713012251371349

**Published:** 2025-08-25

**Authors:** Tamara Backhouse, Anne Killett, Yun-Hee Jeon, Eneida Mioshi

**Affiliations:** 1 School of Health Sciences, 6106 University of East Anglia, Norwich, UK; 2 Sydney Nursing School, The University of Sydney, NSW, Australia

**Keywords:** advanced dementia, interviews, meaning making, care home, family carer

## Abstract

People with cognitive impairment such as those with dementia, particularly in the moderate or advanced stages, can be excluded from qualitative interview research. However, the value of people with dementia’s participation in research is increasingly acknowledged. Adaptations to qualitative interview techniques for use with people with advanced dementia are underexplored. We draw on 13 semi-structured interviews undertaken with people with advanced dementia in family and care-home settings. We examine researcher intuitive adaptations to contextualise and clarify including the importance of member checking in the moment with direct, closed questions if need be; people with dementia ‘coasting’ and leading; as well as carers as conversation partners. We draw out learning points around creating safe interview interactions, and strategies to enhance meaning making. We present methodological learnings and considerations when interviewing people with advanced dementia likely to be instructive for other researchers. We suggest that the research topic needs to be relatable and multiple methods of meaning making should be considered. Our analysis contributes to the growing evidence base considering how to improve meaningful engagement with people with dementia in interviewing. We encourage other researchers to reflect on their interview interactions with people with dementia to further delineate successful components and inform future research practices.

## Introduction

### Including People with Advanced Dementia in Research

The later stages of dementia are characterised by significant losses in daily function and often include considerable impairments in communication and understanding (Mitchell, 2015), consequently dementia is a leading condition for dependency in everyday life. Traditionally research routinely excluded people with dementia in favour of including carers or proxies or only included those in the milder stages with capacity, who could communicate well and provide informed consent without proxy involvement (Shephard et al., 2019; Taylor et al., 2012). Exclusion could be due to researchers considering extra ethical approval and consenting processes ‘too hard’ or time consuming, their perception that this group cannot voice their experiences reliably (Brooks et al., 2017; Killett et al., 2023), and/or gatekeepers such as family carers or care-home staff deciding people with dementia would not want to take part (Reis et al., 2020). This may be a paternalistic view where ethical protocols are valued more than the potential insights from people with dementia (Rochford-Brennan et al., 2019). This skews any evidence base away from the self-reported experiences and needs of this group, which adds to the challenge of effectively shaping care, support, or services.

Despite this backdrop, accounts directly from people with dementia have been gaining prominence over the last decade for example, via Dementia Diaries (Dementia Diaries, 2023) and the Dementia Engagement and Empowerment Project (DEEP), a network of dementia voices in the UK (DEEP, 2023). These and other initiatives empower people with dementia to share experiences and be heard. The need to include, rather than exclude, people with dementia in research is also increasingly recognised (Cridland et al., 2016; Gebhard & Mir, 2021; Hellström et al., 2007; Samsi & Manthorpe, 2020).

### Interviewing People with Dementia

Perspectives of people with dementia about how they want to be involved in service development and research are increasingly known and reported. Some recent examples include the Open Doors Project (DEEP, 2023), Scottish Dementia Working Group (Alzheimer Scotland, 2025), the neighbourhoods and dementia study (Neighbourhoods and Dementia Study, 2025). There is also increasing emphasis on inclusion in dementia research that has been evident particularly over the past 5–10 years. This includes inclusion in co-design (Wang et al., 2019); the application of innovative methods many of which use interviewing as core to their approach (Phillipson & Hammond, 2018) and the long history of ethnography in care settings (which frequently combines interviewing with observation) (For example, Chatwin et al., 2022).

Interviews give participants opportunity to express themselves, providing their commentary on their experiences. Yet, interviewing people with advanced dementia can place pressure on the researcher to navigate relatively unchartered territory in data collection. To ensure data generation with people with cognitive, thinking and communication difficulties such as dementia is adequate, adaptations to traditional data collection processes are needed. However, in qualitative research quality indicators such as long detailed responses and the interview as a ‘self-reported’ account (Kvale & Brinkman, 2008) are closely linked with rigour (trustworthiness and dependability) (Johnson et al., 2020). People with advanced dementia are unlikely to be able to provide lengthy or comprehensive answers or articulate their thoughts cogently, therefore, researchers need to support them.

Key contextual factors to be considered include making sure interviews take place in an acceptable, familiar, and safe context and at a preferred time of day; consent and/or assent are sought; there is enough time; relationships and rapport are developed prior to the interview, and if needed knowledge about the person can be gained from carers (Dementia Enquirers, 2019; Digby et al., 2016; Hellström et al., 2007; Murphy et al., 2015; Nygård, 2006). Additionally, during interviews, researchers should be patient and empathetic, use listening skills, be willing to follow the participant, use repetition if needed, and avoid factual questions requiring memory to answer to reduce possible distress if they cannot answer the question or recall the event (Cridland et al., 2016; Murphy et al., 2015; Nygård, 2006). Observations (and other data collection methods) combined with interviews can enhance researcher knowledge of the person and add trustworthiness and dependability through enabling triangulation (Casey et al., 2016). The role of aids such as TalkingMats©, picture symbols as communication aids; vignettes to access participant’s opinions; written, photo or audio diaries; observations (Bartlett, 2012); projective techniques including word association and hypothetical photographs (Allam et al., 2023; Gresham et al., 2021); walking interviews (Bartlett & Brannelly, 2019); field notes, or carer support can augment or facilitate data collection processes (Nygård, 2006; Samsi & Manthorpe, 2020). Additionally, while exploring perspectives and experiences of people with dementia is important to advance the knowledge and understanding that underpins care practices, research participation needs to be ethical and acceptable for people with advanced dementia (Samsi & Manthorpe, 2020).

### Meaning Making when Interviewing People with Advanced Dementia

Researchers have found interviews with people with dementia often elicit short answers (Gebhard & Mir, 2021; Murphy et al., 2015), simultaneously their attention may not last very long, and they may become fatigued making it difficult to hold a long enough conversation to elicit deep meaning. Therefore, there is heightened onus on the researcher to adequately probe without making the interaction too hard for people with dementia, and to generate meaning in short time periods. Researchers need to expect to assist the person through their interview, being creative and supporting meaning making while also avoiding leading the participant and controlling the story (Nygård, 2006).

Meaning making is often portrayed as the creative component of qualitative analysis (Hunter et al., 2002). Here, we refer to meaning making during data generation, particularly how we may foster it when interviewing people with advanced dementia. We take the position that meaning making is an active process during interviews (Holstein & Gubrium, 2003). Meaning is inescapably collaboratively constructed as the product of the interaction between researcher and interviewee (Garfinkel, 1967; Holstein & Gubrium, 2003), with potential benefit of a support person present. Both interview interactional procedures and the content of the information communicated contribute to meaning making (Holstein & Gubrium, 2003). In addition, meaning can be implicit in participant responses and researchers should make efforts to attend to this for example by reflecting their perception of implicit meanings back to interviewees during interviews (Kvale & Brinkman, 2008). In the moment meaning making could be particularly important for people with dementia due to difficulties with memory. Barbour and Schostak (2005) suggest that researchers address power imbalances to create authentic meaning making situations during interviews. They advise not objectifying the person, listening carefully, and matching the participant’s language and demeanor. However, how this may work sensitively with people with dementia so as not to be odd or patronising is unclear.

In this article we aim to reflect on examples of interviewing people with advanced dementia to contribute to the growing evidence base as to how to improve meaningful engagement with people with dementia in interviewing.

## Methods

### People with Dementia’s Experiences of Assistance with Personal Care

The Pro-CARE programme of research (2018–2022) aimed to investigate care assistance for people with advanced dementia, and care refusals. People with advanced dementia were the focus of the research as their care needs are higher. The mixed methods programme of research involved:

• **Informant-based measures** regarding neuropsychological symptoms, agitation, functional ability, comorbidities, professional input, and environment modifications were filled in with family carers or care-home staff to find out the factors associated with refusals of care (Backhouse et al., 2023; Backhouse et al., 2022).
• **Interviews** with people with advanced dementia, family carers and care-home staff to examine experiences of providing and receiving care (Backhouse et al., 2022).
• **Video-recorded observations** of personal care interactions to examine interpersonal dynamics (Backhouse et al., 2024).

The programme of research aimed to develop learning to feed into educational resources for carers. This article focuses on interviews with people with advanced dementia undertaken within a constructivist paradigm.

Ethics and data collection: Ethical approval for the research programme was received from the Queen’s Square Research Ethics Committee in the UK (IRAS ID: 251339 /REC reference: 18/LO/1677). People with advanced dementia were included in the informant-based measure stage if they were assessed as having advanced dementia with the Frontotemporal dementia Rating Scale (FRS) (Mioshi et al., 2011). The FRS is a well validated 30-item dementia staging tool, completed through a caregiver interview, assessing changes in areas such as completing household chores, selfcare, finances, and behaviour. Total score is the percentage of applicable scores where no change was identified compared to a participant’s premorbid function. Advanced dementia is categorised for scores 40% and below. Lower scores indicate greater dementia severity. People with dementia were aged 65 or over and received physical assistance with their personal care, they either lived in their family home or a care home. A care partner (family carer or care-home staff) who physically assisted with the person’s personal care was recruited for each person with dementia to make a dyad. Participants taking part in the informant-based measure stage had the option of taking part in a qualitative interview and/or observation/s. For interviews, people with dementia were required to be able to verbally communicate. For each dyad, the informant-based measure stage took place before the interviews and in a few cases, participants had also taken part in observations prior to the interviews. After the informant-based measure stage, participants led the scheduling and order of any further data collection. Recruitment of care-home residents was via care-home managers. Recruitment of people with dementia supported at home was via flyers to community dementia services or by contacting those previously signed up to research databases.

In line with the Mental Capacity Act (2005), the lead author assessed the capacity of potential participants to consent to take part in the research through conversations focussed on determining whether the person could understand the information about the research decision, retain and weigh up the information for decision making, and communicate their decision. For potential participants lacking the capacity to consent to take part in the research, personal consultees (family members, friends) were asked to provide advice as to whether they thought the person with dementia would have been likely to want to take part in the interview had they have had the capacity to consent themselves. Where it was advised that the person with dementia would likely have wanted to take part, and the person could communicate verbally (for example, express needs and preferences) and agreed to take part in a recorded interview, they were included in the study. Out of 73 potential participants, 13 interviews took place. The main reason for interviews not taking place was due to a decision by the researcher and/or consultee (often both) that the person would not manage the interview due to understanding or communication difficulties, three did not take place due to changes in circumstances, and three were lost to follow up.

### Data Collection

Interviews were inductive and exploratory. They took place in the person with dementia’s own home or care home at a place they were comfortable with (kitchen, living room, bedroom), they were semi-structured (a topic guide was created with input from family carers, care-home staff, and a care-home resident) and audio-recorded. Interviews focussed on the person’s experiences of receiving help with everyday activities, including likes and dislikes. To increase emotional comfort, care partners were included in the interview or present during the interview, or not, as preferred by participants. Care partners participating in interviews had consented.

The researcher (a senior postdoctoral dementia care researcher with extensive experience as a paid care worker in care homes) already had substantial information about the person with dementia from the informant-based measure stage. Interviews took place on the researcher’s second visit when some rapport with the person and care partner had been developed (Cridland et al., 2016) and, in some cases due to participant preference, observations had taken place shortly before. Care partner advice was sought on the best way to communicate with the person with dementia. When interviewing people with dementia, the researcher took note of non-verbal communication to assess whether the person was happy to participate and continue participating. A study-specific distress protocol was used where the researcher would pause, assess the situation and/or withdraw if they or the care partner noted any signs of distress, uncomfortableness, or fatigue (verbal or non-verbal cues) from the person with dementia. Care partners were encouraged prior to data collection to communicate any instances of distress they noticed. Interviews were recorded and transcribed verbatim.

### Analysis

The analysis of verbatim transcripts focussed on people with dementia’s experiences of receiving assistance with personal care. Inductive qualitative content analysis was used (Elo & Kyngas, 2008). This approach involves immersion in the data, open coding, creating categories and using abstraction to generate generic categories. The analysis process enabled a space for reflections on meaning making, the researcher role and engagement of the person with dementia. Leading on from these reflections, for this article, the transcripts were re-examined in relation to interaction processes, the first author who conducted the interviews re-read all transcripts and the second author read a subset of transcripts, both attending to meaning making (including how the researcher attempted to elicit meanings through the interactions, what and how the person with dementia was supported by, and the person with dementia’s perceived experience) between the researcher and the person with dementia. Notes were made and a meeting between all authors took place to discuss data and draw out learning from the interactions. After further engagement with transcripts, areas of significance in relation to the data generation and meaning making with people with advanced dementia were examined, reflected on, and considered by all authors. We draw on key examples to explore learning for researchers.

## Findings: Key Considerations when Interviewing People with Advanced Dementia

The findings are based on semi-structured interviews with 13 people with dementia (six people supported in their own home, seven care-home residents). Seven participants were female. Across participants, total interview time was 2 hr, 29 min, range 2.18–27.27 min (mean 11.57).

Reflecting on meaning making processes two themes were generated: (1) contextualisation, and (2) the researcher role: adapting, leading, and following. Through these means we demonstrate the value of interviewing people with advanced dementia and our learnings about the adaptations necessary for the researcher to inhabit.

### Contextualisation

Previous data collection via observations of personal care activities for some dyads/people with dementia assisted the researcher during interviews. Observational experiences could be drawn on to examine participant’s experiences, for example:

Care-home resident 7: Shortly after hair washing and foot spa observations (P: person with dementia; R: researcher)

R: …so if you could tell me what it’s like when they’re [carers] helping you wash your hair?

P: It’s strange really. Erm, you get your hair very, very wet and their hands are fiddling about on you and pushing and that and you know they get it, roughing around, getting it very clean and very, very much that way.

…

R: You put your feet in the foot spa over there [points to foot spa]. What was that like?

P: A bit funny as it were, unusual and no, it’s alright. You know it’s being clean and it’s easier than, got a shoe on it or anything like that. What was it? Brushing it, it would be a brush, wouldn’t it?

Here the researcher used the recent experiences observed to draw out the participant’s thoughts, this worked to orientate the person to the activity, so they could describe how it was for them. The foot spa, which was mentioned halfway through the interview, was used as a visual aide-mémoire. By drawing on actual instances of care shortly after them taking place, the participant could articulate the sensory experience of the interaction as it felt to them, without the need to recall and use specific vocabulary.

Care partners were present during ten of the thirteen interviews (two were joint interviews) and had a vital role in supporting the participant to engage in the process. Having care partners present could be an advantage, enabling the person to articulate their thoughts. For example, during this interview:

Person supported at home 5 (P: person with dementia; C: care partner; R: researcher)

P: Sometimes I get a bit iffy, don’t I? [to care partner]

C: Yeah.

P: But yeah.

R: What do you mean by iffy?

C: Cross!

P: Cross!

R: [laughs] so what makes you cross?

P: Me!

The care partner offered the word ‘cross’ which the person with dementia then repeated with emphasis and adopted for themselves. This enabled the researcher to go on to access the reason for getting ‘iffy’ which was the person themselves in terms of their dementia. The interjection from the care partner worked to help the person with dementia to articulate their feelings, perhaps demonstrating a shared knowledge which enabled the researcher to find out more.

Care partners also facilitated the interview process by bringing the person in and helping them understand the interview questions. For example,

Person supported at home 6 (P: person with dementia; C: care partner; R: researcher)

R: And what is it like for you [person name] when [care partner] is helping you with washing your hair?

P: I don’t know. Do you wash your hair? [joking voice]

C: Wash your hair!

P: [laughs]

C: When I wash your hair.

P: My hair.

C: What does it feel like?

P: Awful. [joking voice]

R: [laughs]

C: I don’t think you’re going to get …serious.

R: [laughs] No!

P: I come out there awful, drips and all that I mean.

C: Yeah well.

P: Oh God!

C: You don’t mind, do you?

P: I’d rather go back to bed!

C: You don’t mind.

P: [laughs]

R: Is there any way you particularly like it to be done, your hair washing?

P: Well I’m told I’ve got to do it and that’s it.

C: [laughs]

P: It’s not quite so bad, you know, do I like it? That’s got to be done.

C: [laughs]

…

C: Your toenails.

P: There’s no toenails in there, is there? [laughs]

C: Yeah well, we have a go with the toenail clippers every now and again, don’t we? [to person with dementia]

P: Well yeah, we do. Ooh! I have to, I have to, when I start getting that sort of, that things out, I start to screw up in a heap! [laughing voice]

R: [laughs] aw, so not your favourite?

P: No [laughs].

In these excerpts the person with dementia was joking about their experience and thriving on the interaction, continuing to take their turn as a conversation partner in the interaction. Initially, little information was gained about the person’s experience of having their hair washed as they deflected the question back. This appeared to have been a key strategy for some people with advanced dementia, which we term here as coasting, meaning to hold their turn in the interaction until they could respond to the question, or due to the question being too open. The participant had fun with the interaction and then started to disclose aspects of their experience with hair washing. The care partner brought in the person with dementia, which worked to move the dialogue along, facilitating and opening the way for the person’s description of their experience and feelings. The mood was light throughout. In the second section, the interaction between the two worked well in relation to toenail cutting, enabling the researcher to ascertain the person with dementia’s feelings about the care activity. The researcher checked their interpretation of the person with dementia’s words in the moment (*not your favourite?*) without having to access this information through an account of difficulty, which could have taken the person to a place of distress in retrieving it. Taking part in interviews appeared to be enriching for many participants, in this example, the participant with dementia appeared to enjoy the interaction, was happy to be involved, and made jokes throughout.

### The Researcher Role: Adapting, Leading, and Following

The researcher role in eliciting the experiences of people with advanced dementia was critically important. A semi-structured topic guide was employed more loosely than it would have been in interview settings with people without cognitive impairment, since adapting to the participant in real time became the main resource required of the researcher. For example, the interview may have not been long enough to cover all topics dependant on the participant’s fatigue, or some topics emerged in the context of the interview to be too complex for some participants. This highlighted the importance of the researcher knowing the topic guide and flexibly adapting as needed.

Researcher decisions in the moment were crucial. If the question was too open or complex, there was a sense of setting the person up to fail. In the following excerpt an open question appeared too vague for the participant, and they took the dialogue to an area where they could contribute. This participant strategy was present in a few interviews, particularly when the question was cognitively demanding. This is another example of a person with dementia using coasting, a strategy they used to maintain their part in the conversation by taking the dialogue into an area they were able to talk about, which as often reported in dementia is a reminiscence from much earlier in their life.

Care-home resident 1 (P: person with dementia; R: researcher)

P: They [carers] help me here, they have done from the word go.

R: Mm what sort of things do they help you with? *[question too vague for participant]

P: Well I’m just looking, that was the [name of train]. They got on the thing; well, my grandfather was driving that!

R: Goodness [surprise as a famous train].

P: Yes. And he used to grab me to help him.

R: So I just came and watched you and [care partner] a little moment ago with your teeth and doing your shave. *[researcher draws on observation]

P: Yes.

R: How was that for you? *[question still too vague]

P:

R: How do you find being helped? *[question more specific]

P: I’m not used to it. [laughs] *[author additions to demonstrate]

Researcher flexibility was key, adapting with the next question to offer the participant more guidance and direction in the interaction. This required following the person while also preparing to adapt the next question making it more focussed and specific to lead the participant to an understanding of what the researcher was asking about. Through each interaction component, the researcher gradually determined the level of leading required for each participant enabling contributions about their experiences of receiving assistance with personal care activities. Validating the person’s response and experience [*goodness*] was vitally important to maintain psychological wellbeing in interviews. There were tricky decisions to make around how much to validate and follow the participant and how soon to redirect to the interview topic.

A lot of additional effort was focused on meaning making within the interviews with people with dementia. The following example (Part 1) shows how it was necessary for the researcher to adapt the flow and focus to check interpretations in the moment.

Part 1: Person supported at home 1 (P: person with dementia; R: researcher)

P: As a man, before this, when I went to the toilet, I went straight to the toilet, now [with an incontinence pad] I’ve got to go [makes noise and gestures].

R: Ah, do you mean you’ve got so much to take off. Yeah.

P: [inaudible] And if you did go, aargh.

R: So you find the pad a bit frustrating?

P: More than a bit.

R: And do you find it a bit helpful as well…?

P: No they get in the way.

The researcher checking interpretation of meaning in the moment was vital to aid understanding of the participant’s experiences. Particularly when communication was partial, and answers were short. Often, meanings were only implied, though sometimes partly created with augmentative and alternative devices, such as gesture (Teachman et al., 2018). In the above excerpt the researcher starts to use closed questions to pin down the participant’s thoughts about incontinence pads. The researcher was interpreting the person’s responses but wanting to check these interpretations were correct as the information given was not explicit. Where the participant articulated “*aargh*” the researcher checked that this was a representation of frustration. However, to check out meanings, the researcher necessarily strayed into the possibility of leading the person towards a certain interpretation. A further closed question was used to double check interpretation (“*helpful as well?*”) and verify if alternative meanings were also valid. The interaction reflects a departure from traditional open question, long participant dialogue interviewing style and could be interpreted as poor interviewing. However, in the context of communication difficulties, structured support was often required to get to the perspective of the participant. This structured support enabled the researcher to make explicit the implicit by collaboratively revealing the participant’s meaning.

Generally, participants were keen to get their perspectives across. For example, the same participant led the interview to an area he felt was pertinent, even though the care partner was not encouraging:

Part 2: Person supported at home 1 (P: person with dementia; C: care partner; R: researcher)

P: It [bowel] doesn’t work the same as it would do as normal because in the medicines, they make them [inaudible]

C: No, you’ve become constipated…

P: I know and what I’m just telling you is what that means to me.

R: Mm.

C: No.

P: It hardens, let me finish [to care partner]! Then you blow it down in flames!

C: Aw [sound of despair]

P: For God’s sake, it’s life, that keeps it moist, otherwise it wouldn’t go through your body.

….

R: No thank you [to person with dementia]. For me to learn about what it’s like for you is really important so thank you, that’s useful.

P: Extremely important. I like to [inaudible] so I’m not in it.

This care partner found it difficult when the person with dementia led the interview to their experience with constipation and bowel movements. As we have shown, joint interviews or interviews with care partners present mostly worked to facilitate elicitation from the person with dementia. However, here, the person with dementia had to push to get their point across while the care partner found it uncomfortable, they perhaps felt their partner was being disinhibited to bring this up. The participant was determined to communicate his experience, and the researcher enabled this by listening intently and validating the importance of the message afterwards. This demonstrates the empowerment an interview situation can provide for a person with dementia and the way they can lead the direction of an interview. Following the participant, if the person were able to lead, worked to enable key experiences to be examined and meanings revealed. Of note, not many interviewees would have been able to lead the dialogue in this way.

## Discussion

Reflections on interview dialogues with people with advanced dementia have provided insight into ways in which researchers can hear their stories. People with advanced dementia appeared to enjoy interview interactions and had important things to say about their experiences. Meaning making was generated through: (1) the researcher eliciting the experiences of people with advanced dementia by being flexible and making decisions in the moment; validating, fluctuating the level of leading/following in response to cues, member checking (checking interpretation of meaning), and adapting for example from open to closed questions; (2) the person with dementia responding, articulating, coasting, and rising to the occasion, and (3) care partners inviting the person in to the conversation, drawing out experiences, and providing correct words. Recent researcher/person with dementia interaction experiences and visual cues supported interactions and therefore meaning making.

### Meaning Making during Interviews with People with Advanced Dementia

**Figure 1.**
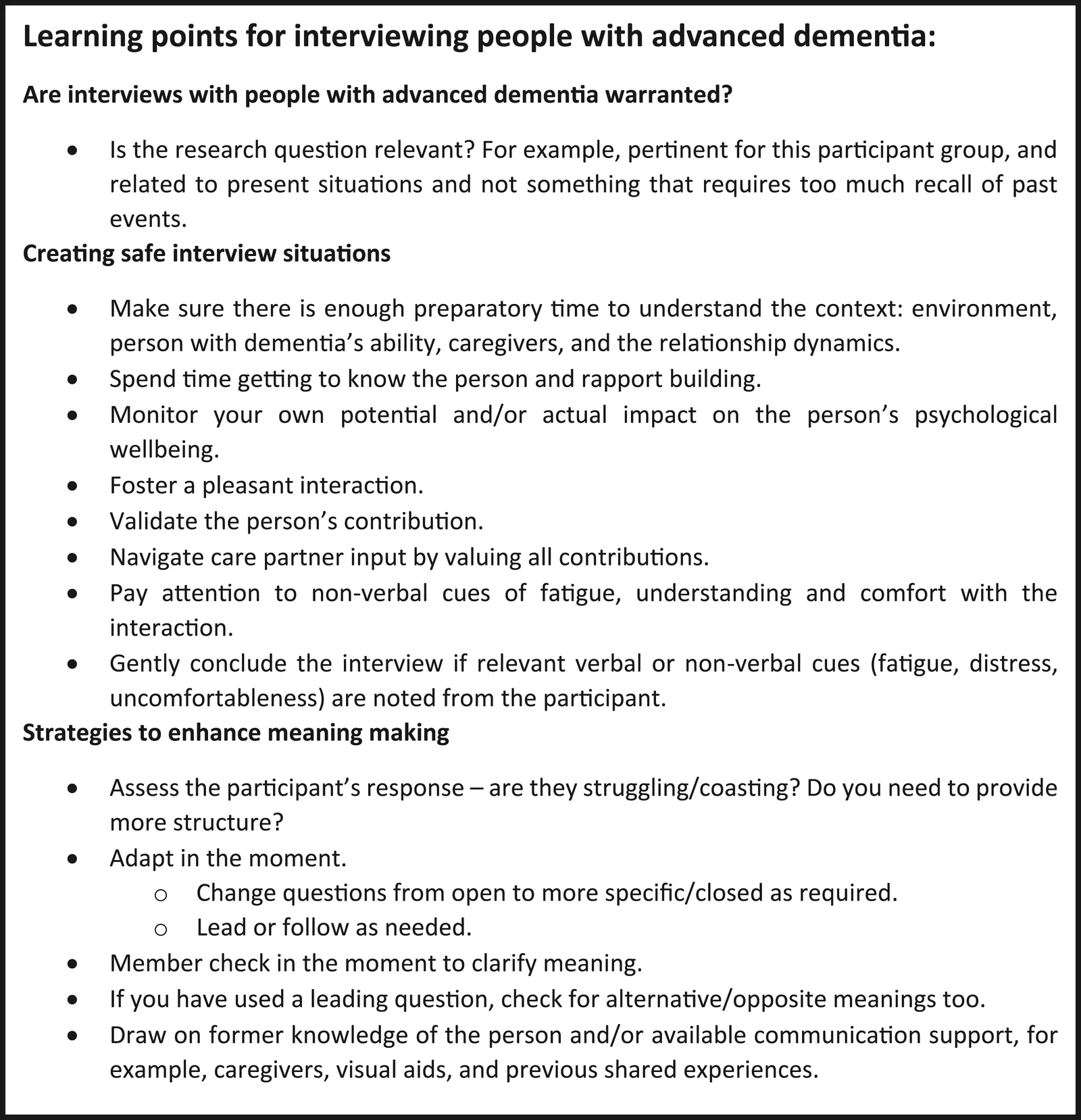
Learning Points for Interviewing People With Advanced Dementia

Meaning making is collaboratively constructed throughout interview interactions between the researcher and participant (Holstein & Gubrium, 2003), with the role of the care partner often facilitating further engagement. Concerns that family or carer presence could influence or monopolise responses from people with dementia (Zarhin, 2018) were mitigated in our study through the researcher having a clear focus on the person’s own story (Birt et al., 2020). Through reflecting on the active process of meaning making within interviews, we have delineated learning points for researchers interviewing people with advanced dementia (see Figure 1), which are likely to also be important when interviewing those with moderate dementia. Effective meaning making can only occur in safe interactions (Hellström et al., 2007). We found strategies advised by Barbour and Schostak to be useful. For example, we listened carefully through being attentive and responsive, employed terms used by participants, and made sure to treat and view each person in a way that valued their individuality and humanity. However, we did not try to match participants’ manners and ways of being (Barbour & Schostak, 2005), as this could be inappropriate with this participant group. Instead, emphasis was placed on matching participants’ cognitive and communication abilities, where necessary, with structured support provided through more specific and focussed questions and explicitly checking meaning.

Learning points necessitate that researchers are attuned to signals from the person and adeptly adapt in the moment. The need for tailoring of conversation supports has been previously highlighted (Phillipson et al., 2019). We found, taking time to understand the data collection context (communication cues, previous observations, care partners) and build rapport with participants prior to and during interviewing was key (Cridland et al., 2016). Rapport building with people with advanced dementia who may not recall a previous interaction included the researcher drawing on former knowledge. For example, by using information gained from conducting the informant-based measure and/or observation stages, the researcher could adapt to the person’s abilities and talk about things they knew were relevant or important to them. Previous research encounters were advantageous for supporting rich interviews with people with advanced dementia.

The need for the researcher to adapt (Nygård, 2006) by providing more information, using closed and specific questions for clarification when perceived as needed, and leading the person could lead to a lack of credibility in the data. Consequently, reflexivity is needed concerning the balance between leading the participant and obtaining data where meaning can be interpreted (Lloyd et al., 2006). We advise checking out meanings and possible counter meanings in the moment as participants are often not able to revisit moments of meaning later. This process was not just productive for validating or invalidating researcher interpretations of implicit meanings, the unsaid, (Kvale & Brinkman, 2008), but more generally to seek clarity where communication was partial, gestured, or unclear.

Humour has been highlighted before as a mechanism to maintain relationships and offset stressors in dementia (Hickman et al., 2018). In our study, the use of humour appeared to work in three ways: (1) to build (further) rapport between the person with dementia and the researcher, (2) to enable people with dementia to be able to talk about the potentially embarrassing topic of personal care assistance, and (3) to enable the person with dementia to ‘rise to the occasion’ enjoying speaking with a different person who was interested in their story.

Researcher skills learnt through past experiences interacting with people with dementia and qualitative interviewing are likely to be useful when interviewing people with advanced dementia (Murphy et al., 2015). To conduct ethical interviews, researchers must be open and attuned to each person and communicate with emotional sensitivity (Digby et al., 2016). The ability to read non-verbal or verbal cues and respond flexibly is vital (Nygård, 2006). For example, care partners may have the necessary communication skills that can be drawn on. Additionally, being comfortable with semi-structured interviewing processes for example, actively listening and adapting the order of questions during the interview to enhance flow is essential (Nygård, 2006). In this study, the researcher collecting data had many years’ experience as a care worker and was a practiced qualitative researcher and the wider research team had considerable expertise both methodologically and clinically as occupational therapists (AK, EM) and a nurse (YHJ). Researchers interviewing people with advanced dementia are likely to need training and/or experience in both methodological and clinical aspects.

Our reflections add to knowledge for including people with advanced dementia in interview studies by examining ways usual modes of interviewing were adapted in the moment and how they elicited meaning from participants. We have set out productive, practical ways to generate and check insightful data from this group. Previous work has highlighted using repetition, taking time, following the participant, and avoiding factual questions when interviewing people with dementia (Cridland et al., 2016; Murphy et al., 2015; Nygård, 2006). Our study endorses these strategies and shows closed questions and leading the participant may also be important, provided they are accompanied by in the moment member checking. Additionally, observation may be a useful way of using facts to elicit further meaning without relying on participant recall. As found previously (Gebhard & Mir, 2021; Murphy et al., 2015), our participants also provided short answers to questions. However, researcher perception of participant fatigue (Hellström et al., 2007), ability to contribute further, and comfort with the interview process were the main drivers for short interview lengths, demonstrating that short interviews should be expected with this population. For these decisions, the researcher drew on their clinical experience as a care worker where they had honed skills in reading cues and clues and adapting to residents. When interviews were finished, the researcher thanked the participant, stopped the recorder, and had a pleasant chat about the person’s interests or cues in the environment, checking they were okay before ending the interaction.

### Findings from the Topic Focussed Analysis

The knowledge gained from interviewing people with advanced dementia in this study was valuable and insightful. From our topic-focussed analysis of interview data, three themes were generated regarding people with advanced dementia’s perspectives about receiving assistance with personal care: (1) personal care assistance as a sensory experience, (2) adjusting to changing abilities, and (3) perceiving the caregiver role (see supplemental material). These key insights will feed into the development of an intervention to improve personal care interactions. Our research had a practical and current focus: it was a good fit for the use of the interview method with this group compared to an abstract topic.

### Strengths and Limitations

Our study enabled the perspectives of people with advanced dementia to be heard in relation to receiving assistance with personal care. The interview process, including building rapport with the person prior to the interview, seemed to empower people with dementia by providing them with the opportunity and means to disclose their experiences, which they appeared to become more confident to do. Interviews were one-time events; therefore, the study may have benefitted by using multiple interviews with each participant to gain an accurate picture of their experiences over time. Using visual resources such as objects or photographs during interviews may also have been helpful. In those interviews where care partners were present, their presence may have made people with dementia more guarded in their responses, however predominantly, our data suggests care partners facilitated data collection and meaning making. Due to the focus on people with advanced dementia, many of the potential participants taking part in the informant-based measure stage were unable to take part in an interview, this limited our sample size. Those taking part could often only be interviewed for a short duration, due to communication difficulties or fatigue, which limited the volume of data collected. Where communication was impaired, even with checking, we may not have accurately interpreted meanings.

## Conclusions

Interviews producing insightful knowledge can be conducted with people at the advanced stages of dementia. However, enabling true participation meant flexibly adapting to changes related to their condition, often necessitating deviations from open question interviewing techniques to focus on meaning making with participants. The role of caregivers/study partners can be invaluable in supporting contributions from people with advanced dementia. We encourage other researchers to reflect on their interview interactions with people with dementia to further delineate successful components and inform future research practices.

## Supplemental Material

Supplemental Material - Principles and Strategies of Interviewing People With Advanced Dementia Supplemental Material for Principles and Strategies of Interviewing People With Advanced Dementia by Tamara Backhouse, Anne Killett, Yun-Hee Jeon, and Eneida Mioshi in Dementia.

## Data Availability

The data that support the findings of this study are available from the corresponding author, [TB], upon reasonable request.*
