# Supplementary material for: Principles and Strategies of Interviewing People with Advanced Dementia
Source: Dementia (London). 2025 Aug 25;25(7):1512–29. doi: 10.1177/14713012251371349 (PMC13569913; doi:10.1177/14713012251371349)
Supplement: Supplementary file 1 [file sj-pdf-1-dem-10.1177_14713012251371349.pdf]

SUPPLEMENTARY Material

Table 1: Findings from people with advanced dementia: *experiences of people with dementia receiving assistance with personal care*

Themes	Subthemes	Illustrative quotes
**Personal care assistance as a sensory experience**	**Bodily sensations**	**Care-home resident 7**<br>*P – Oh she's a damn (sic) sight better than I am [laughs], you know what I mean. They rough it on there, they rough it. They're doing, I think they're, you know they're doing it a better way because a) they get them, so they clean so much more. I mean they do a proper job.*
	**Preferences for care**	**Care-home resident 1**<br>*P - …it's nice to feel clean err you know nice clean clothes on. No, I don't like feeling sweaty.*
**Adjusting to changing abilities**	**Emotionally managing the need for assistance**	**Person supported at home 3**<br>*R - Why does it irritate you?*<br>*P – Erm because I'm normally self-sufficient.*<br>*R – Mm.*<br>*P – And I find it irritating that I'm not self-sufficient anymore.*
	**Adapting with new routines**	**Person supported at home 1**<br>*P- I've been told to go the toilet like women do, sit on it*
	**Continuities of self**	**Person supported at home 2**<br>*P - It's stupid isn't it but you see my mum right from*

		when I can remember we always used to have a strip in the morning… you know it was just ritual, just ritual. …you do it automatically; you are not washed if you don't do it properly!
**Perceiving the caregiver role**	**Caregiver connection during interactions**	**Care-home resident 3**<br>*P – No I like things like that, it's done, when you're chatting to each other, I think so.*
	**Acceptability of the caregiver approach**	**Care-home resident 2**<br>*P - They're [carers] generally helpful but some are pressingly helpful. You know and I say "Go."*
	**Concerns over caregivers' experiences of personal care**	**Person supported at home 3**<br>*P – She [wife] empties it [catheter bag]. Erm with a jug and she's very brave, I think.*<br>*R – Why is that?*<br>*P – Because she smells the urine.*<br>*R – Ah okay.*<br>*P – And I think that's not a nice thing for anybody to smell.*
	**Ongoing relationships**	**Care-home resident 1**<br>*P - When I first came here, I thought 'I don't like her' [carer] but when you get to know her, she's lovely, she*

will do anything for you. ... I wouldn't like a stranger to do it, no and she makes fun of it, you know there's always something that she does that makes you laugh.

P: Person with dementia; R: Researcher
